# Impact of Li_3_BO_3_ Addition on Solid Electrode-Solid Electrolyte Interface in All-Solid-State Batteries

**DOI:** 10.3390/ma14227099

**Published:** 2021-11-22

**Authors:** Evgeniya Il’ina, Svetlana Pershina, Boris Antonov, Alexander Pankratov

**Affiliations:** Institute of High Temperature Electrochemistry, Ural Branch, Russian Academy of Sciences, 20 Akademicheskaya St., 620990 Ekaterinburg, Russia; svpershina_86@mail.ru (S.P.); B.Antonov@ihte.uran.ru (B.A.); a.pankratov@ihte.uran.ru (A.P.)

**Keywords:** all-solid-state lithium-ion batteries, solid electrolytes, interface, LiCoO_2_, Li_4_Ti_5_O_12_

## Abstract

All-solid-state lithium-ion batteries raise the issue of high resistance at the interface between solid electrolyte and electrode materials that needs to be addressed. The article investigates the effect of a low-melting Li_3_BO_3_ additive introduced into LiCoO_2_- and Li_4_Ti_5_O_12_-based composite electrodes on the interface resistance with a Li_7_La_3_Zr_2_O_12_ solid electrolyte. According to DSC analysis, interaction in the studied mixtures with Li_3_BO_3_ begins at 768 and 725 °C for LiCoO_2_ and Li_4_Ti_5_O_12_, respectively. The resistance of half-cells with different contents of Li_3_BO_3_ additive after heating at 700 and 720 °C was studied by impedance spectroscopy in the temperature range of 25–340 °C. It was established that the introduction of 5 wt% Li_3_BO_3_ into LiCoO_2_ and heat treatment at 720 °C led to the greatest decrease in the interface resistance from 260 to 40 Ω cm^2^ at 300 °C in comparison with pure LiCoO_2_. An SEM study demonstrated that the addition of the low-melting component to electrode mass gave better contact with ceramics. It was shown that an increase in the annealing temperature of unmodified cells with Li_4_Ti_5_O_12_ led to a decrease in the interface resistance. It was found that the interface resistance between composite anodes and solid electrolyte had lower values compared to Li_4_Ti_5_O_12_|Li_7_La_3_Zr_2_O_12_ half-cells. It was established that the resistance of cells with the Li_4_Ti_5_O_12_/Li_3_BO_3_ composite anode annealed at 720 °C decreased from 97.2 (*x* = 0) to 7.0 kΩ cm^2^ (*x* = 5 wt% Li_3_BO_3_) at 150 °C.

## 1. Introduction

All-solid-state batteries attract considerable scientific attention because such batteries have a number of advantages over commercially produced lithium-ion batteries, including increased safety, a wider operating temperature range, increased resistance to an aggressive atmosphere and high pressures, greater stability in the case of battery depressurization, and long lifetime [1,2,3,4]. According to the literature data [5,6,7,8,9], Li_7_La_3_Zr_2_O_12_-based solid electrolytes are attractive lithium-ion conductors for all-solid-state lithium and lithium-ion power sources. Li_7_La_3_Zr_2_O_12_ (LLZ) solid electrolyte has two structural modifications—tetragonal (I41/acd) and cubic (Ia-3d). The cubic modification is of greatest interest as a solid electrolyte for power sources, since its lithium-ion conductivity at room temperature (10^−3^–10^−4^ S cm^−1^) is 2–3 orders of magnitude higher compared to the tetragonal one [9,10]. However, the introduction of a dopant (for example, Al, Ga, Y, Nb, Ta, etc.) is required for stabilization of the highly conductive cubic LLZ [9]. Nevertheless, the high resistance at the solid electrode–solid electrolyte interface is one of the critical issues that should be addressed for mass production of all-solid-state power sources [3,4,9,11,12,13].

The research into the cathode–solid electrolyte interface optimization is still in its early exploratory stage. In some studies, the use of buffer layers, for example, Li_3_PO_4_ [14,15], LiPO_3_ [16], Li_2_SiO_3_ [17], Li_3_BO_3_ [18,19,20], Nb [21], etc. is proposed. Moreover, composite cathodes can also be obtained using additives in the form of ionic liquids [22], polymers [23], gels [24], low-melting lithium-containing additives [18], Li(CF_3_SO_2_)_2_N electrolytic salt [25], and lithium-conducting electrolytes [18,26,27]. Nowadays, LiCoO_2_ (LCO) compounds are widely used as a cathode material for lithium-ion batteries due to their high electrochemical characteristics and good cyclability [28]. In the work [18], a low-melting Li_3_BO_3_ additive (25 wt%), which has a lithium-ion conductivity of 2 × 10^−6^ S cm^−1^ at 25 °C, was added to the lithium cobaltite to solve the contact problem between electrode and electrolyte. The cathode material was obtained by the screen-printing method followed by annealing at 700 °C for one hour. K. Park et al. [20] used a mixture of LiCoO_2_ and Li_3_BO_3_ as a cathode with Li_6.06_Al_0.20_La_3_Zr_2_O_12_ solid electrolyte, which was preheated at 700 °C. It was noted that such modification of the cathode material led to a tighter contact at the interface between the electrode and the solid electrolyte, and also prevented the chemical interaction between LiCoO_2_ and Li_7_La_3_Zr_2_O_12_ with the formation of a low-conductivity La_2_Zr_2_O_7_ phase during heat treatment and cell cycling. In addition, more complex in composition (multicomponent) cathode materials are used in all-solid-state power sources [19,29]. For example, In_2(1−*x*)_Sn_2*x*_O_3_, Li_3_BO_3_ and polyvinylidene fluoride (PVDF) were introduced into LiCoO_2_ [20] and Li[Ni_0.5_Co_0.2_Mn_0.3_]O_2_ [29] cathode materials. A significant decrease in the resistance at the cathode–solid electrolyte interface based on LLZ was observed, which in turn led to a decrease in polarization resistances and, as a consequence, to an improvement in the electrochemical characteristics of the all-solid-state battery in terms of capacity and Coulomb efficiency. It was also noted [19] that an increase in temperature from room values to 80 °C leads to a decrease in the total resistance of the cells studied. It should be noted that the available literature contains no data about the influence of Li_3_BO_3_ additive amount and the heat treatment conditions on the resistance at the cathode–solid electrolyte interface.

Lithium titanate Li_4_Ti_5_O_12_ (LTO) is considered to be a promising anode material for lithium-ion batteries due to its high theoretical capacity—175 mA h g^−1^, low degradation during cycling, and small volume change of the unit cell during intercalation/deintercalation of lithium ions [30]. It can be argued that LTO does not degrade during battery operation, unlike other anode materials (amorphous silicon, carbon/graphite, lithium metal and its alloys) [31,32]. In the work [33], it was shown that thin LTO films deposited on Li_6.25_Al_0.25_La_3_Zr_2_O_12_ ceramics using pulsed laser deposition demonstrated stable operation during cycling with capacity values close to the theoretical one. The interface optimization between LTO and Li_6.25_Al_0.25_La_3_Zr_2_O_12_ can be achieved through interface engineering: in this case, a stepwise electrolyte–electrode transition is created by introducing the anode into the porous layer of the electrolyte sample [34]. The modified cells had lower resistance values and improved capacitive characteristics compared to traditional electrode deposition (casting). In the work [35], LTO|Li_6.25_Al_0.25_La_3_Zr_2_O_12_|Li all-solid-state cells with a capacity of 70–75 A h kg^−1^ were assembled. It was established that capacity growth and the formation of optimal interface between solid electrolyte and electrode can be realized by isostatic pressing of the cells during their assembly. Yoshima et al. [36] introduced a 2 wt% polyvinylidene fluoride (PVdF) binder and 3 wt% addition of PAN-based monomer into the anode material in order to increase the contact area between the Li_7_La_3_Zr_2_O_12_ solid electrolyte and LTO. We propose another method of solid electrolyte–electrode interface optimizing by the sintering process of powdered LTO with a low-melting additive.

The choice of Li_3_BO_3_ (LBO) as an additive in the composite electrode creation is dictated by the fact that it has the lowest melting point and makes it possible to create dense protective coatings in a composition with more refractory compounds. Li_3_BO_3_ is a lithium-ion conductor and its coating can increase the concentration of Li^+^ in the contact layer with the solid electrolyte [37]. The aim of this work was to study the effect of LBO addition on the interface processes and total resistance of half-cells LCO/LLZ and LTO/LLZ.

## 2. Materials and Methods

Li_2_CO_3_,La_2_O_3_,Al(NO_3_)_3_ × 9H_2_O and ZrO(NO_3_)_2_ × 2H_2_O were used as starting components for the sol-gel synthesis of the cubic modification of Li_7_La_3_Zr_2_O_12_ with 0.15 mol of Al_2_O_3_ (c-LLZ). La_2_O_3_ was pre-dried at 1000 °C to a constant weight. The reagents were mixed in the stoichiometric ratio, except Li_2_CO_3,_ which was taken with the excess of 10 wt%, as demonstrated in [9,10]. Lanthanum oxide and lithium carbonate were dissolved in diluted nitric acid. ZrO(NO_3_)_2_·2H_2_O and C_6_H_8_O_7_·H_2_O were dissolved in a small amount of distilled water. The solutions obtained were mixed and evaporated to a transparent gel at 80 °C. Then, the gel was dried and heated at ~200 °C. The synthesis was performed by increasing the temperature stepwise (700 °C—1 h; 800 °C—1 h; 900 °C—1 h). The samples of solid electrolytes were cold-pressed into pellets at 240 MPa and sintered in air for 1 h at 1150 °C.

Li_2_CO_3_, Co(NO_3_)_2_ × 6H_2_O, and C_6_H_8_O_7_ × H_2_O were used as the starting materials for obtaining the LiCoO_2_ by sol-gel synthesis as demonstrated in [38]. Lithium carbonate was dissolved in diluted nitric acid. Co(NO_3_)_2_ × 6H_2_O and C_6_H_8_O_7_ × H_2_O were dissolved in a small amount of distilled water. The solutions obtained were mixed and evaporated to a gel. Then, the gel was dried and heated at ~200 °C. The resulting product was annealed in air at temperatures of 500 and 700 °C for one hour.

Li_4_Ti_5_O_12_ was synthesized by sol-gel synthesis using Li_2_CO_3_ (analytical grade) and tetraethoxytitanium (C_2_H_5_O)_4_Ti (pure grade) as demonstrated in [39]. Sol-gel synthesis was carried out with citric acid C_6_H_8_O_7_ (reagent grade) as a complexing agent. The hydrolysis of a preset amount of tetraethoxytitanium at a ratio of Li:Ti = 4:5 was carried out on a magnetic stirrer with heating for three hours in a glassy carbon cup, followed by dissolution of a white precipitate of metatitanic acid with the addition of diluted HNO_3_ (1:1, extra pure grade). As a result, a transparent solution of titanyl was prepared, to which a solution of Li_2_CO_3_ with citric acid was added (the optimal ratio of citric acid R to the total amount of metal ions was 1/2, which was previously determined in [30]). As a result, a clear solution was obtained, which was evaporated to form a gel at 80 °C for twelve hours. Then the gel was heated in air to a temperature of ~200°C and held for five hours. Upon subsequent heating to 500 °C and holding for one hour, all organic compounds were completely decomposed and volatilized. Then the resulting blend was sintered in an Al_2_O_3_ crucible at 750 °C for one hour, at 800 °C for five hours in air. After the end of each regime, the mixture was ground in an agate mortar for thirty minutes.

Li_3_BO_3_ was obtained via a standard melt quenching method [40,41]. Starting components such as Li_2_CO_3_ and H_3_BO_3_ were mixed in the stoichiometric ratio and annealed at 1100 °C for thirty minutes in a Pt crucible. Then the melt was quenched between two stainless steel plates.

The thermal behavior of mixtures consisting of c-LLZ, LiCoO_2_, Li_3_BO_3_ or Li_4_Ti_5_O_12_ was investigated using simultaneous thermal analysis (STA). The STA measurements were performed in the Pt pans with a heating rate of 10 °C min^−^^1^ in air at an expulsion rate of 20 mL min^−^^1^ in the temperature range of 35–800 °C utilizing a thermal analyzer Netzsch STA 449 F1 Jupiter (Netzsch, Selb, Germany). The results obtained were processed by the NETZSCH Proteus software.

LiCoO_2_- and Li_4_Ti_5_O_12_-based composite electrodes with different Li_3_BO_3_ additions (0–15 wt%) were obtained by spraying from isopropanol slurry. The powders of electrode material with glass additive taken in an adjusted ratio were thoroughly mixed with isopropanol (99.9%) on a magnetic stirrer for twenty-four hours. The resulting suspension was sprayed onto heated to 100 °C pellet of preliminarily ground c-LLZ by Ultra airbrush (Harder&Steenbeck, Norderstedt, Germany). The half-cells were dried at 100 °C and then annealed at 700 and 720 °C for thirty minutes.

The phase composition of the synthesized solid electrolytes, electrode powders, and electrode composites, with different LBO content after heat treatment at different temperatures, was investigated by X-ray diffraction analysis (XRD). XRD was performed with a Rigaku D-MAX-2200V diffractometer (Rigaku, Tokyo, Japan) with a vertical goniometer at Cu K_α_-radiation and 2θ = 10–60°. The identification of compounds was carried out using a PDF-2 database (2009).

The cross-section of LiCoO_2_ + Li_3_BO_3_|c-LLZ and Li_4_Ti_5_O_12_ + Li_3_BO_3_|c-LLZ half-cells was investigated by scanning electron microscopy (SEM) using a TESCAN MIRA 3 LMU (TESCAN, Brno, Czech Republic). SEM images in SE (secondary electrons) and BSE (back-scattered electrons) modes were obtained at a high voltage of 10 kV and beam intensity of 10 mA.

Impedance measurements of GaAg|(100 − *x*)LiCoO_2_ + *x*Li_3_BO_3_|c-LLZ|GaAg and GaAg|(100 − *x*)Li_4_Ti_5_O_12_ + *x*Li_3_BO_3_|c-LLZ|GaAg cells were conducted in the air atmosphere using an immittance meter E7-25 (MNIPI, Minsk, Belarus) in the frequency range of 0.0251000 kHz in a two-probe cell with silver electrodes at temperatures from 25 to 340 °C. The cathode material completely covered the ceramic sample surface on one side, and a gallium-silver paste (GaAg) was used as the electrode on the other side. To check the reproducibility of the results, conductivity measurements were performed on several sets of samples.

## 3. Results and Discussion

### 3.1. Determination of Heat Treatment Conditions for LiCoO_2_- and Li_4_Ti_5_O_12_-Based Composite Electrodes

The stability of the cubic Li_7_La_3_Zr_2_O_12_ doped by Al in contact with LiCoO_2_ was evaluated in our previous work [38]. It was established that no exothermic or endothermic peaks are observed on the DSC curve after heating up to 900 °C. Moreover, the phase composition of the c-LLZ and LiCoO_2_ mixture does not change after heating at 400, 600 and 800 °C according to the XRD data. So, it was concluded that there is not any interaction between the solid electrolyte and the electrode material. The melting point of Li_3_BO_3_ was determined by DSC and the possible interaction of a mixture of c-LLZ, LCO, and Li_3_BO_3_ up to 800 °C was estimated.

**Figure 1:**
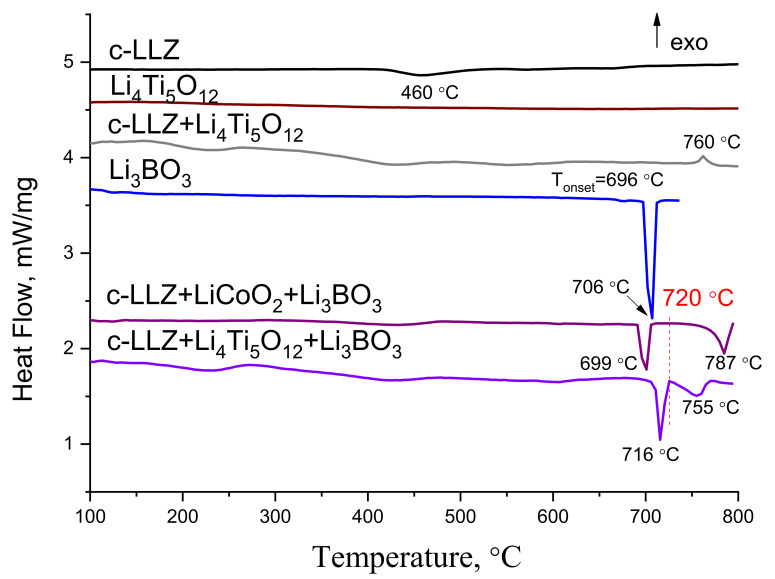
DSC curves of c-LLZ, Li_3_BO_3_, Li_4_Ti_5_O_12_ and mixtures of c-LLZ + LCO + Li_3_BO_3_, c-LLZ + LTO, c-LLZ + LTO + Li_3_BO_3_ powders.

DSC curves of individual materials (c-LLZ, Li_3_BO_3_, Li_4_Ti_5_O_12_) and mixture of powders (c-LLZ + LCO + Li_3_BO_3_ (1:1:1), c-LLZ + LTO (1:1), c-LLZ + LTO + Li_3_BO_3_ (1:1:1)) are shown in Figure 1. An intense endothermic peak at 706.6 ± 1.5 °C is associated with Li_3_BO_3_ melting, which is in good agreement with the literature data [42]. The second endothermic peak at 787 °C is presumably related to the interaction in the c-LLZ + LCO + LBO mixture. So, based on the data obtained, 700 and 720 °C were chosen as the temperatures for sintering electrode materials to the c-LLZ solid electrolyte.

### 3.2. LiCoO_2_/Li_3_BO_3_ Composite Cathode

**Figure 2:**
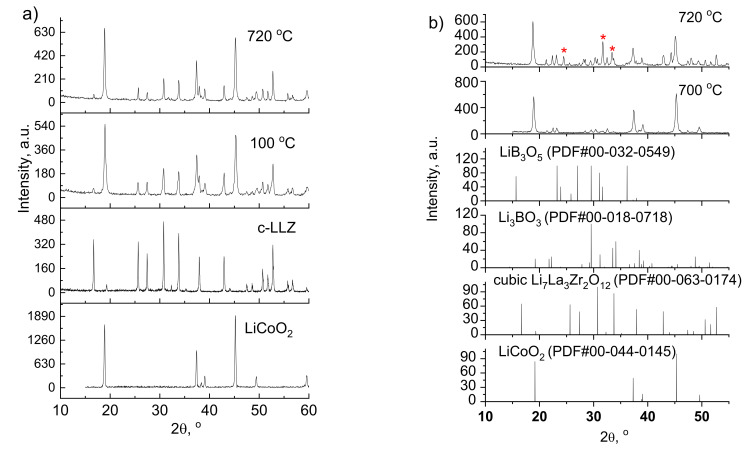
XRD patterns of LiCoO_2_ (**a**) and LiCoO_2_ + 5 wt%Li_3_BO_3_ composite cathode (**b**) after sintering onto c-LLZ substrate at different temperatures. *—La_2_Li_0.5_Co_0.5_O_4_ (PDF#01-083-1842).

LiCoO_2_ was sprayed onto the c-LLZ surface and then annealed at 100, 700 and 720 °C. According to the XRD data, the heat treatment of half-cells up to 720 °C does not lead to the formation of any impurity phases, Figure 2a. Peaks of lithium cobaltite and c-LLZ are found in the diffraction patterns. The presence of peaks related to cubic modification of LLZ can be explained by X-ray penetration due to the thin layer of the deposited cathode material. Then, (100 − *x*)LiCoO_2_ + *x*Li_3_BO_3_ composite cathodes with different contents of low-melting additive (*x* = 5,10 and 15 wt%) were deposited on the surface of the solid electrolyte and annealed at 700 and 720 °C. According to XRD data, the phase composition of the composite cathode does not depend on the content of lithium borate. Besides the main phases (LCO, LBO and c-LLZ), additional peaks of LiB_3_O_5_ were observed in the XRD patterns of half-cells annealed 700 °C. The annealing temperature growth (up to 720 °C) leads to the formation of La_2_Li_0.5_Co_0.5_O_4_ impurity phase. In the work [43], a thin layer of La_2_CoO_4_ (~50 nm) was observed at the LLZ|LiCoO_2_ in the assembled all-solid-state battery. Moreover, the possibility of this impurity phase formation was established using thermodynamic simulation in our previous work [44]. Since the appearance of this impurity was not identified by the XRD method in the cell with pure LCO, it can be assumed that the low-melting addition of lithium borate promotes this interaction.

**Figure 3:**
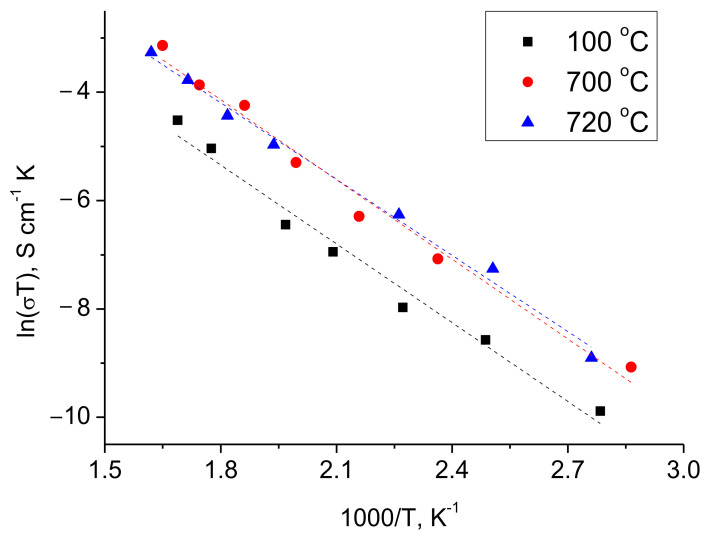
Arrhenius plots for the total conductivity of LiCoO_2_|c-LLZ half-cells annealed at different temperatures.

It should be noted that LCO powder without lithium borate addition poorly held onto ceramics after drying at 100 °C, while annealing at higher temperature led to a tighter contact between the electrode and the solid electrolyte. These data are confirmed by the results of the half-cells resistance measuring. The temperature dependences for the conductivity of LCO|c-LLZ half-cells annealed at different temperatures are shown in Figure 3. It can be seen that there is an increase in the conductivity of the half-cells after temperature growth from 100 to 700 °C. However, a slight increase in temperature from 700 to 720 °C does not lead to further conductivity growth.

**Figure 4:**
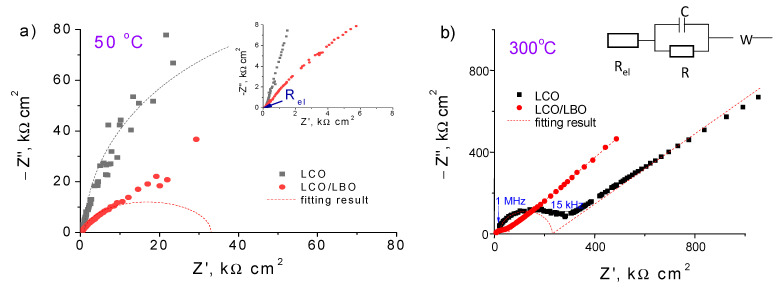
Impedance plots of LiCoO_2_|c-LLZ and LiCoO_2_ + 5 wt% Li_3_BO_3_|c-LLZ half-cells at 50 (**a**) and 300 °C (**b**).

**Figure 5:**
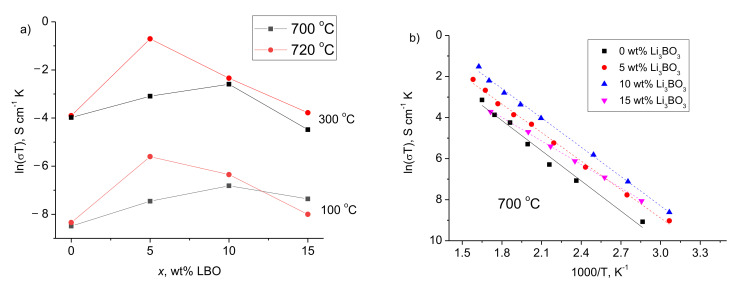
Concentration dependences (**a**) and Arrhenius plots (**b**) for the total conductivity of (100 − *x*)LiCoO_2_ + *x*Li_3_BO_3_|c-LLZ half-cells.

The typical impedance plots of LCO|c-LLZ and LCO/LBO|c-LLZ at 50 and 300 °C are presented in Figure 4. The total resistance of the half-cells was determined from the intersection of the arc and low frequency tail with the real part of the impedance value Z′. At close to room temperatures, the resistance values of the half-cell with lithium cobaltite could not be established due to the high interface resistance, Figure 4a, while the 5 wt% Li_3_BO_3_ addition with subsequent heat treatment at 720 °C led to a significant decrease in the interface resistance at the cathode–solid electrolyte even at 50 °C. It can be seen that one semicircle is visible, but it does not come out of zero, Figure 4a. It was found that the resistance value between zero and the point of the semicircle beginning refers to the resistance of the c-LLZ since these values are in good agreement with the values measured separately for the solid electrolyte. Figure 4b also shows an equivalent circuit, according to which the total cell resistance is the sum of the resistance of the electrolyte and the resistance at the interface between the electrode and the solid electrolyte. According to the data obtained, the additive content as well as the heat treatment temperature of the half-cells affect the interface resistance between the solid electrolyte based on the Li_7_La_3_Zr_2_O_12_ and LCO/LBO composite cathode. The optimal conditions for interface resistance decrease were reached using composite cathode with 5 wt% Li_3_BO_3_ addition annealed at 720 °C, Figure 5a. Apparently, the decrease in the sintering temperature for this composite cathode leads to a smaller contact area between cathode particles and ceramic electrolyte. To ensure a tight contact, either a larger glass addition is required (10 wt% LBO, Figure 5a,b) or a longer exposure time of sintering should be applied. Thus, a decrease in the interface resistance from 260 to 40 Ω cm^2^ at 300 °C is observed when a composite cathode with 5 wt% Li_3_BO_3_ is applied, in comparison with pure lithium cobaltite.

**Figure 6:**
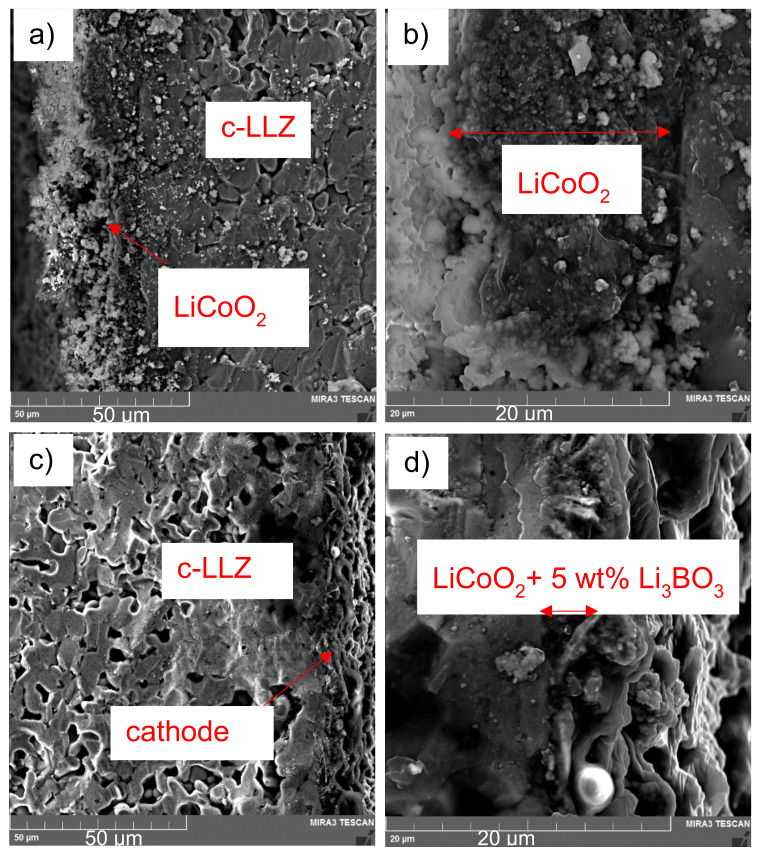
SEM images of the cross-section of LiCoO_2_|c-LLZ (**a**,**b**) and LiCoO_2_ + 5 wt% Li_3_BO_3_|c-LLZ (**c**,**d**) half-cells, after heating at 720 °C.

SEM images of the cross-section of LCO|c-LLZ and LCO + 5 wt% LBO|c-LLZ half-cells after heating at 720 °C are shown in Figure 6. It can be seen that the cathode material without LBO addition presents clearly visible particles of lithium cobaltite. However, the morphology of the cathode material significantly changes after the addition of low-melting LBO. The cathode has a less loose structure and better contact with the ceramics.

### 3.3. Li_4_Ti_5_O_12_/Li_3_BO_3_ Composite Anode

**Figure 7:**
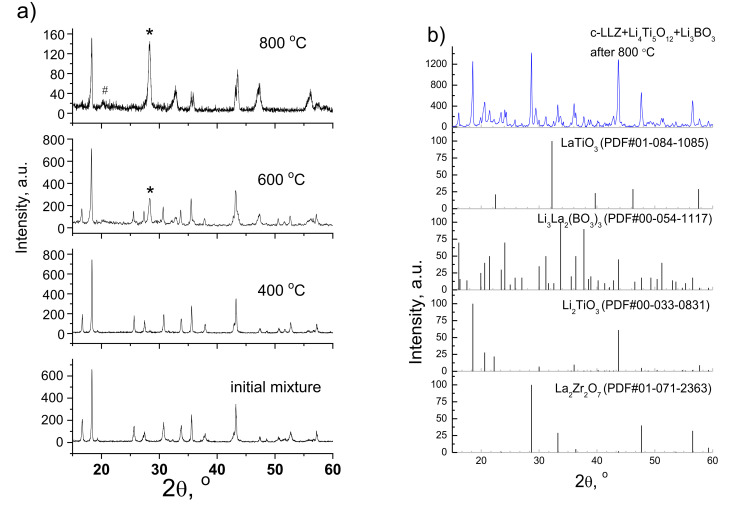
XRD patterns of Li_4_Ti_5_O_12_ + c-LLZ (1:1) mixture annealed at different temperatures (**a**) and c-LLZ + Li_4_Ti_5_O_12_ + Li_3_BO_3_ (1:1:1) annealed at 800 °C (**b**). *—La_2_Zr_2_O_7_, #—Li_2_TiO_3_.

DSC curves of LTO and c-LLZ mixture with the same weight ratio were investigated to identify the possible products of their interaction, Figure 1. The endothermic peaks at ~250 and ~430 °C can be referred to as the removal of adsorbed water and CO_2_ from the c-LLZ sample, respectively [45,46]. The endothermic peak at ~588 °C can be referred to the removal of lithium oxide leading to La_2_Zr_2_O_7_ formation on the solid electrolyte powder surface, the reflections of which can be detected in the XRD patterns of the LTO and c-LLZ mixture annealed at temperatures above 600 °C, Figure 7a. The endothermic peak at 760 °C is probably related to chemical interactions between the components. Therefore, the XRD analysis of the mixture studied was additionally carried out after annealing at higher temperatures (800 and 900 °C). Li_2_TiO_3_ and La_2_Zr_2_O_7_ impurity phases are detected, Figure 7a.

The addition of Li_3_BO_3_ to the mixture studied leads to the appearance of additional endothermic peaks at 716 and 755 °C on the DSC curve, which are related to lithium borate melting and components interaction, respectively. The chemical interaction of the components investigated is confirmed by XRD data. The reflections from Li_2_TiO_3_, La_2_Zr_2_O_7_, LaTiO_3_, and Li_3_La_2_(BO_3_)_3_ can be observed in the XRD patterns of c-LLZ + LTO + Li_3_BO_3_ (1:1:1) mixture annealed at 800 °C, Figure 7b. Based on the data obtained, the temperatures of 700 and 720 °C were chosen for sintering the (100 − *x*)LTO/*x*Li_3_BO_3_ composite anode to the c-LLZ electrolyte surface.

**Figure 8:**
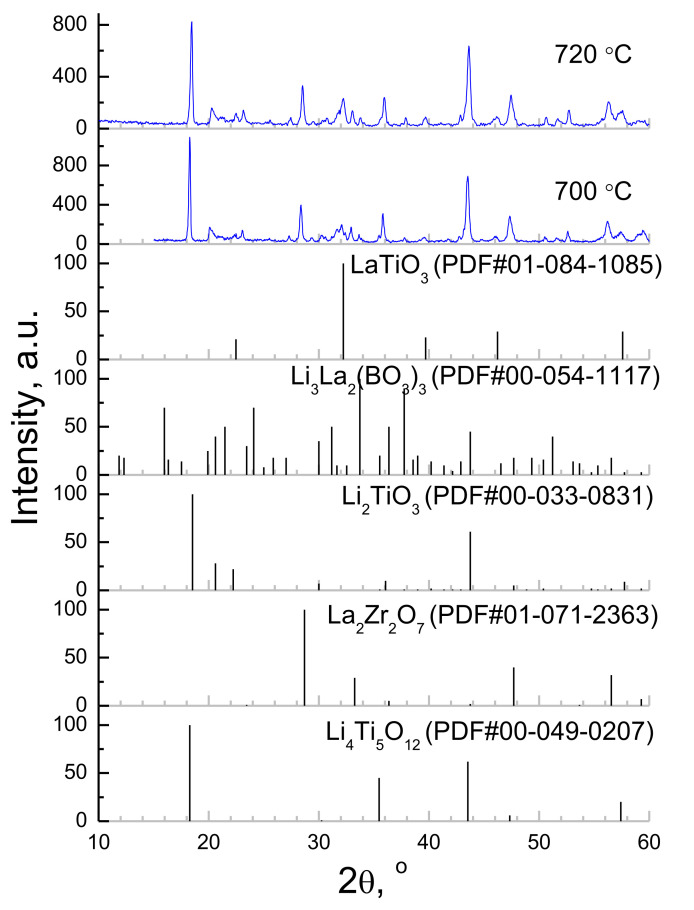
XRD patterns of Li_4_Ti_5_O_12_+5 wt%Li_3_BO_3_ composite anode after sintering onto c-LLZ substrate at 700 and 720 °C.

XRD patterns of the surface of LTO/LBO|c-LLZ half-cells after heat treatment at 700 and 720 °C are shown in Figure 8. Li_2_TiO_3_, La_2_Zr_2_O_7_, LaTiO_3_, and Li_3_La_2_(BO_3_)_3_ impurity phases in addition to the main phase of Li_4_Ti_5_O_12_ are observed in Figure 8. Their formation is related to isothermal holding of the half-cells at ≥700 °C for 0.5 h, in comparison with the DSC study which was carried out with a constant heating rate without holding. Thus, the lithium borate introduction leads to the appearance of additional phases at high sintering temperatures of LTO with c-LLZ. Similar behavior was observed during the heat treatment of the Li_1.5_Al_0.5_Ge_1.5_(PO_4_)_3_ solid electrolyte with a LTO/LBO composite anode [47].

**Figure 9:**
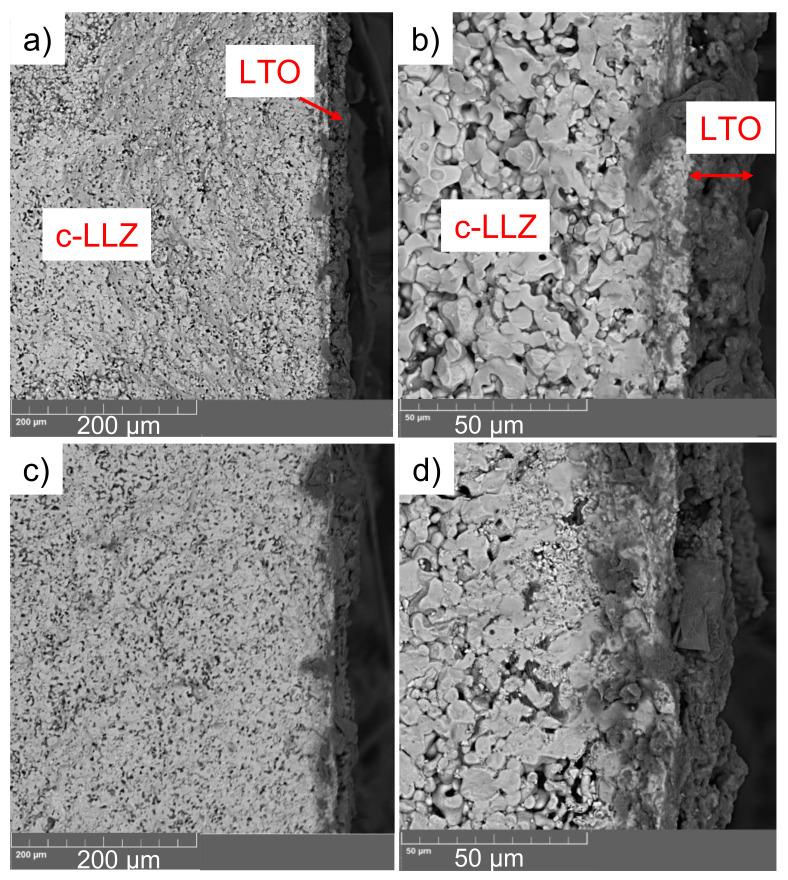
SEM images of the cross-section of Li_4_Ti_5_O_12_|c-LLZ (**a**,**b**) and Li_4_Ti_5_O_12_ + 5 wt% Li_3_BO_3_|c-LLZ (**c**,**d**), after heating at 720 °C.

**Figure 10:**
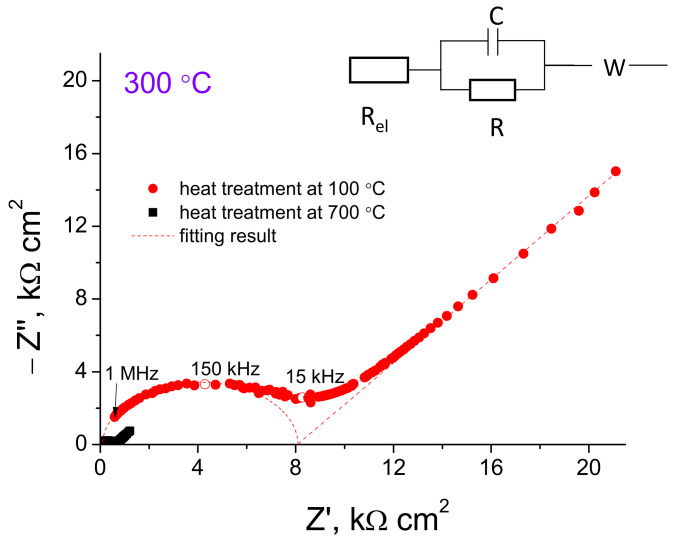
Impedance plots of Li_4_Ti_5_O_12_|*c*-LLZ half-cells after heating at 100 and 700 °C.

**Figure 11:**
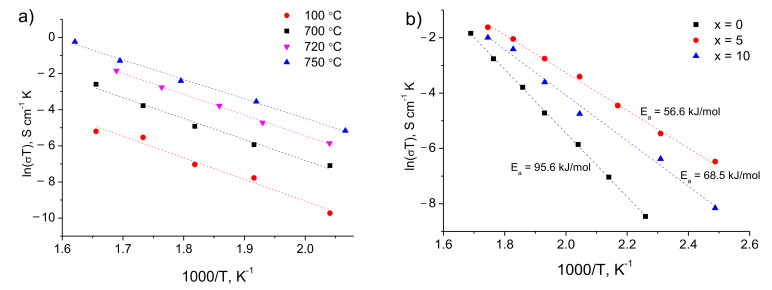
Arrhenius plots for the total conductivity of half-cells: (**a**) Li_4_Ti_5_O_12_|c-LLZ after heat treatment at different temperatures (100, 700, 720 and 750 °C); (**b**) (100 − *x*)Li_4_Ti_5_O_12_ + *x*Li_3_BO_3_|c-LLZ, annealed at 720 °C.

As can be seen from the micrographs, Figure 9, the introduction of LBO leads to an increase in the contact of LTO particles with c-LLZ. The impedance data for the LTO|c-LLZ and LTO/LBO|c-LLZ half-cells were collected across a wide temperature range to estimate the influence of Li_3_BO_3_ addition on the interfacial resistance between anode material and solid electrolyte. The impedance plots present a semicircle that does not come to a zero point, and a low frequency tail; from their intersection with the real part of the impedance value, the total resistance of the half-cells was determined. The high resistance values of the studied half-cells are caused by the interface resistance. The increase in the sintering temperature of Li_4_Ti_5_O_12_|c-LLZ half-cells from 100 to 750 °C leads to a decrease in the total resistance by two orders of magnitude, despite impurity phase formation (La_2_Zr_2_O_7_) during heat treatment, Figure 10 and Figure 11a.

Despite the fact that the highest conductivity values in LTO|c-LLZ half-cells were reached at 750 °C, the heat treatment temperature of LTO/LBO composite anode was lower than the interaction temperature in the c-LLZ + LTO + Li_3_BO_3_ mixture (725 °C, Figure 1) and was equal to 700 and 720 °C. It was established that the introduction of LBO additive into LTO leads to a decrease in interfacial resistance with solid electrolyte and an increase in specific conductivity of the half-cells studied with a decrease in the activation energy, Figure 11b. The lower resistance values of the cells studied were achieved with the addition of 5 wt% LBO. The total resistance of LTO|c-LLZ cells is equal to 97.2 kΩ cm^2^ while the resistance of LTO + 5 wt% LBO|c-LLZ was 7.0 kΩ cm^2^ at 150 °C. The decrease in resistance can be caused by an increase in the interfacial solid-solid contact during the softening of Li_3_BO_3_ above the melting point. However, the introduction of 10 wt% LBO into LTO leads to resistance growth with the activation energy increase, which is probably associated with an increase in the impurity content. Thus, the interfacial resistance between c-LLZ and the solid electrode can be reduced by obtaining composite electrodes with Li_3_BO_3_ addition.

## 4. Conclusions

In the presented work, the effect of Li_3_BO_3_ addition on the thermal stability, chemical compatibility, and interfacial resistance between cubic Li_7_La_3_Zr_2_O_12_ and electrode materials (LiCoO_2_ cathode and Li_4_Ti_5_O_12_ anode) was investigated. The possible interaction of c-LLZ with LCO, LTO and Li_3_BO_3_ up to 800 °C was studied by differential scanning calorimetry. It was established that the interaction in the studied mixtures with Li_3_BO_3_ begins at 768 and 725 °C for LCO and LTO, respectively. Therefore, 700 and 720 °C were chosen as the sintering temperatures for LiCoO_2_ + Li_3_BO_3_|c-LLZ and Li_4_Ti_5_O_12_ + Li_3_BO_3_|*c*-LLZ half-cells. According to XRD analysis, such heat treatments of LiCoO_2_-based composite electrodes lead to the formation of LiB_3_O_5_ and La_2_Li_0.5_Co_0.5_O_4_ impurity phases. However, a decrease in the interface resistance was observed in LiCoO_2_ + Li_3_BO_3_|c-LLZ half-cells because of Li_3_BO_3_ addition, in comparison with pure lithium cobaltite. According to SEM study and impedance spectroscopy data, optimal contact with the ceramic electrolyte is achieved by using composite cathode with 5 wt% Li_3_BO_3_ addition sintered at 720 °C. Impurity phases of Li_2_TiO_3_, La_2_Zr_2_O_7_, LaTiO_3_ and Li_3_La_2_(BO_3_)_3_ were detected after annealing. However, they do not have a negative effect on the interface resistance of the half-cells studied. According to the data obtained, Li_4_Ti_5_O_12_-based composite anodes with Li_3_BO_3_ addition have the lowest interfacial resistance with the solid electrolyte, which is due to an increase in solid–solid contact. Thus, the optimum amount of low-melting additives and the best possible heat treatment conditions for Li_3_BO_3_-modified composite electrodes based on Li_4_Ti_5_O_12_ and LiCoO_2_ leading to the decrease in the interface resistance with cubic Li_7_La_3_Zr_2_O_12_ were established and can be used in medium-temperature all-solid-state batteries.

## Data Availability

Not applicable.
